# Choosing Wisely: Tailored Drainage Strategies for Peripancreatic Fluid Collections—A Tertiary Center’s Experience

**DOI:** 10.3390/jcm14228018

**Published:** 2025-11-12

**Authors:** Raluca-Ioana Dascalu, Madalina Ilie, Claudiu Stefan Turculet, Bogdan Valeriu Popa, Gabriel Constantinescu, Christopher Pavel, Vlad Rizescu, Cosmin-Viorel Bogu, Teodor Cabel, Oana-Mihaela Plotogea

**Affiliations:** 1 Department of Gastroenterology, Clinical Emergency Hospital of Bucharest, 105402 Bucharest, Romania; raluca-ioana.dascalu@rez.umfcd.ro (R.-I.D.); gabrielconstantinescu63@gmail.com (G.C.); christopher.pavel@gmail.com (C.P.); vladrizescu@gmail.com (V.R.); cosmin-viorel.bogu@rez.umfcd.ro (C.-V.B.); cabelteodor@gmail.com (T.C.); plotogea.oana@gmail.com (O.-M.P.); 2 Department 5, Gastroenterology, Carol Davila University of Medicine and Pharmacy, 050474 Bucharest, Romania; claudiu.turculet@umfcd.ro (C.S.T.); valeriu.popa@umfcd.ro (B.V.P.); 3 Department of Surgery, Clinical Emergency Hospital of Bucharest, 105402 Bucharest, Romania; 4 Department of Radiology, Clinical Emergency Hospital of Bucharest, 105402 Bucharest, Romania

**Keywords:** EUS-guided drainage, lumen-apposing metal stent (LAMS), double pigtail stent (DPS), walled-off necrosis (WON), pancreatic pseudocysts (PPs)

## Abstract

**Introduction**: The management of symptomatic peripancreatic fluid collections (PFCs), including pancreatic pseudocysts (PPs) and walled-off necrosis (WON), remains a clinical challenge. **Methods**: We conducted a single-center retrospective cohort study to compare the efficacy, safety, and cost of endoscopic drainage (lumen-apposing metal stent vs. double pigtail stent) and percutaneous drainage for PFCs. From an initial cohort of 75 patients with symptomatic PFCs between 2020 and 2025, 63 underwent drainage procedures. Primary endpoints were the clinical success, defined as >50% collection size reduction, and the need for direct endoscopic necrosectomy (DEN). Secondary endpoints included adverse events, recurrence rates, length of hospital stay (LOS), and procedural costs. **Results**: In our study, endoscopic drainage proved high clinical efficacy for PFCs, especially PPs. Once a technique was chosen, complication rates were comparable, indicating no clear safety advantage for either approach. While percutaneous drainage relieved symptoms and reduced collection size in half of the cases, the other half had only transient or partial improvement. When comparing endoscopic drainage techniques, median costs and length of hospital stay trended higher for lumen-apposing metal stent (LAMS) than double pigtail stent (DPS), but the differences were not statistically significant. However, the “other” group proved markedly higher costs and the longest mean hospital stay. **Conclusions**: The choice of drainage technique impacts short-term outcomes and safety profile in managing PFCs. Our findings support a tailored, step-up approach, prioritizing endoscopic ultrasound-guided drainage based on PFC characteristics to optimize clinical outcomes.

## 1. Introduction

Peripancreatic fluid collections (PFCs) are well-recognized sequelae of acute pancreatitis, ranging from pancreatic pseudocysts (PPs) to walled-off necrosis (WON) [1]. Although many PFCs regress spontaneously, intervention becomes necessary when collections become symptomatic, infected, or associated with complications such as gastric outlet obstruction, biliary compression, or persistent pain [2,3]. The ideal time for intervention is reported to be at least 4 weeks after pancreatitis onset (the longer the better) to allow the wall to mature, excepting the infection or the mass effect complications [4,5].

Traditionally, management was based on percutaneous or surgical drainage, procedures often burdened by significant morbidity, prolonged hospitalization, and less favorable long-term outcomes. However, over the past two decades, endoscopic ultrasound (EUS)-guided drainage has emerged as the preferred modality, owing to its minimally invasive nature and high clinical efficacy [6]. Advances in stent technology, particularly the introduction of lumen-apposing metal stents (LAMS), have further expanded the role of endoscopy by facilitating direct endoscopic necrosectomy (DEN) in cases of WON [7,8]. In parallel, double pigtail plastic stents (DPS) continue to be widely employed, particularly in the management of uncomplicated pseudocysts, due to their lower cost and familiarity among practitioners [9,10].

Recent literature has compared LAMS with DPS, suggesting that while LAMS might shorten procedure time and enable more efficient drainage of necrotic collections, the safety advantage over plastic stents remains inconclusive [9,11]. Reported complication rates such as bleeding, stent migration, and infection, do not consistently favor one approach [7,8,12,13]. Moreover, cost-effectiveness analyses have produced mixed findings, with some studies indicating higher upfront costs for LAMS that are not always offset by improved outcomes [14]. Beyond endoscopic techniques, percutaneous drainage (PD) retains a role, particularly when endoscopic access is limited; however, its success rates for necrotic collections are modest, and it is often used as part of a step-up approach rather than as definitive therapy [6,15].

In the light of these uncertainties, recent consensus statements advocate for individualized, “tailored” strategies based on the type of collection, patient comorbidities, and local expertise. Nonetheless, real-world evidence comparing the outcomes of LAMS, DPS, and percutaneous drainage remains scarce, particularly in the context of tertiary referral centers where complex cases predominate. To address this gap, we conducted a single-center retrospective cohort study evaluating the efficacy, safety, recurrence, and economic impact of these drainage modalities in patients with symptomatic PFCs. By analyzing short-term outcomes and resource utilization, our study aims to clarify the optimal selection of drainage techniques and to contribute evidence supporting a nuanced, patient-centered approach to PFC management.

## 2. Materials and Methods

**Study design and patient selection**

**Figure 1:**
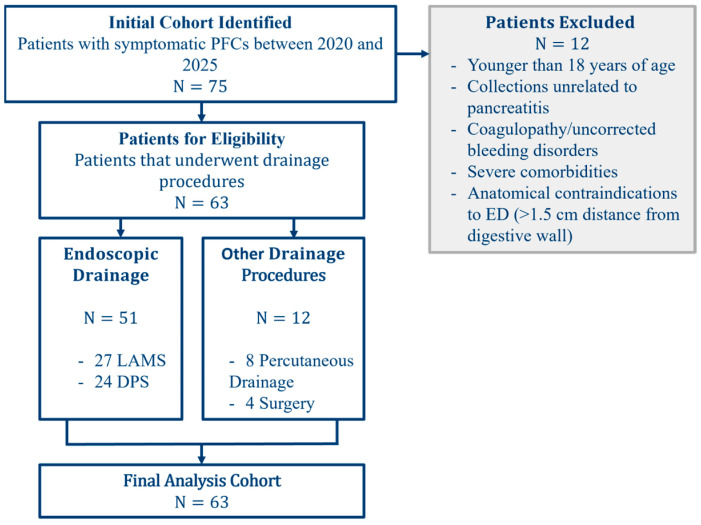
Flow diagram of patients’ selection.

We conducted a retrospective cohort study at the Bucharest Clinical Emergency Hospital, a tertiary referral center, in order to compare the efficacy, safety, and cost of endoscopic drainage (LAMS vs. DPS) and percutaneous drainage or surgery for PFCs. From an initial cohort of 75 patients with symptomatic PFCs between 2020 and 2025, 63 underwent drainage procedures. All consecutive patients with symptomatic PFCs were included. We excluded patients younger than 18 years of age, those with collections unrelated to pancreatitis, and individuals with coagulopathy, uncorrected bleeding disorders, severe comorbidities, or anatomical contraindications to endoscopic drainage (technically unfeasible—e.g., >1.5 cm distance from digestive wall) (Figure 1).

All endoscopic procedures were performed under EUS guidance with a linear echoendoscope. When there were collections with necrotic debris or when direct endoscopic necrosectomy (DEN) was anticipated, we selected lumen-apposing metal stents (LAMS) with sizes of 15 mm × 10 mm or 20 mm × 10 mm, depending on collection size and wall thickness. On the other hand, when we dealt with pseudocysts (PPs) with predominantly fluid content we used double pigtail stents (DPS). For PPs, we routinely used a single 8.5 Fr × 5 cm, while we chose larger caliber or multiple DPS for large collections, necrotic debris, or duct disconnection, and we adjusted length to tract distance.

In several cases (N = 8), we performed percutaneous drainage using a pig-tail catheter of 12 F inserted by the Seldinger technique under ultrasound guidance, using first bougies on the guidewire.

Procedures were performed under deep sedation or general anesthesia by experienced endoscopists.

This study was approved by the ethics committee and registered with the Bucharest Clinical Emergency Hospital. All participants provided written informed consent. Comprehensive access to the study dataset was granted to all authors.

**Outcomes and definitions**

Symptomatic collections were defined by the presence of infection, persistent abdominal pain, gastric or biliary obstruction, or other complications necessitating intervention.

The primary endpoints were: (i) clinical success, defined as a reduction of ≥ 50% in collection size on follow-up imaging and (ii) need for direct endoscopic necrosectomy (DEN) in patients with walled-off necrosis (WON). Secondary endpoints included adverse events (bleeding, stent migration, infection, or perforation), recurrence rates, length of hospital stay (LOS), and procedural costs.

**Data collection and statistical analysis**

Demographic data, type of collection (PPs vs. WON), procedural details, clinical outcomes, and follow-up results were extracted from institutional medical records. Adverse events were categorized according to severity and temporal relation to the intervention. Cost analysis included device costs and hospitalization expenses.

All patients with incomplete clinical or imaging data were excluded from analysis. No imputation methods were applied. Missing follow-up records were handled by case-wise deletion.

Statistical analyses were performed using IBM Statistical Product and Service Solutions (SPSS) software v.26 (IBM Corp., Armonk, NY, USA), Chi-square tests for categorical variables and one-way ANOVA for continuous variables. A *p*-value of <0.05 was considered statistically significant.

## 3. Results

**Baseline characteristics**

A total of 63 patients were included in this prospective study. Among them, 51 (81%) underwent endoscopic drainage of pancreatic fluid collections (PFCs)—27 using LAMS and 24 with DPS—while 12 (19%) were treated with alternative approaches (percutaneous drainage or surgical approach) (Table 1).

Baseline demographic and clinical parameters, including age, gender, size and type of collection, and extent of pancreatic necrosis, were comparable between groups (all *p* > 0.05).

**Figure 2:**
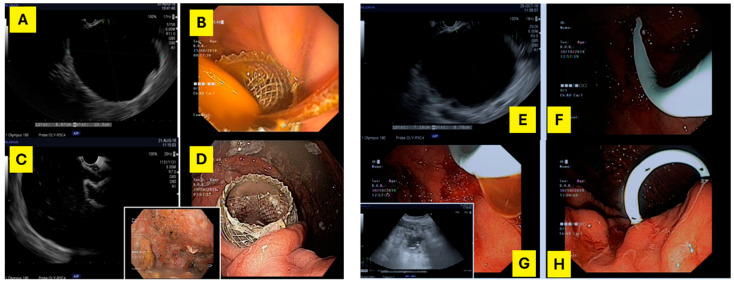
On the left—(**A**,**C**) echoendoscopic image of a 100 mm × 90 mm WON; (**B**,**D**) lumen-apposing metal stents (15 mm × 10 mm) placed transgastrically, draining the WON. On the right—(**E**) echoendoscopic image of a 71 mm × 87 mm pseudocyst; (**F**,**H**)—double pigtail plastic stent (7 Fr × 5 cm) placed transgastrically, draining the pseudocyst; (**G**) echoendoscopic image of residual collection after DPS drainage.

**Figure 3:**
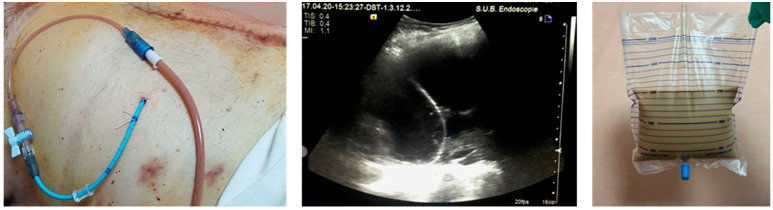
Percutaneous drainage of a 20 cm pancreatic WON (<30% necrosis) evacuating purulent fluid. The drainage was performed using a pig-tail catheter of 12 F inserted by the Seldinger technique under ultrasound guidance, using first bougies on the guidewire.

The mean age of the cohort was 54.8 ± 14.3 years, and the majority were male (69.8%) (Table 1). Regarding the type of collection, 58.7% of patients had PPs, while 41.3% presented with WON. Treatment allocation reflected this distribution: among patients with LAMS (N = 27), the majority (70.4%) had WON; conversely, those treated with DPS (N = 24) were predominantly PPs (91.7%) (Figure 1). The “other” group (N = 12), which included alternative drainage modalities such as PD and surgery, was more evenly distributed between PPs (58.3%) and WON (41.7%) (Figure 2 and Figure 3).

The mean size of collections was 11.5 ± 7.2 cm, with broadly comparable dimensions across groups. Anatomical distribution was varied, with the most frequent locations being the pancreatic body (46%) and body–tail region (30.2%), followed by the head (9.5%), head–body junction (9.5%), and tail (4.8%). Assessment of pancreatic necrosis revealed that necrosis was absent in 22.2%, mild (<30%) in 22.2%, moderate (30–50%) in 28.6%, and extensive (>50%) in 14.3%, with DPS patients more often showing no necrosis and LAMS patients more often showing moderate involvement. The “other” group included the highest proportion of patients with extensive necrosis (>50%; 41.7%) (Table 1).

**Primary and secondary end points**

Treatment selection was strongly driven by the type of collection: 70% of patients who received a LAMS had WON, whereas 88% of those treated with DPS had PPs (*p* < 0.001).

Endoscopic drainage using either LAMS or DPS was associated with significantly higher efficacy, lower complication rates, and shorter hospital stays compared with surgical or percutaneous approaches for the management of PFCs. Mortality and recurrence were infrequent and comparable between groups. Although costs tended to be lower with endoscopic treatment, the difference was not statistically significant (Table 2).

Clinical success, defined as resolution of the collection (reduction > 50%) was achieved in 63% cases, more often in the endoscopic group than in the other interventions (74.5% vs. 25.0%, *p* = 0.002; RR = 2.98, 95% CI 1.10–8.05) and mostly in LAMS group.

The need for endoscopic necrosectomy was reported in 25% of cases, with an average of 2.4 sessions (range 1–6). Concretely, DEN was required in 27.5% of endoscopic cases versus 16.7% of other interventions (*p* = 0.71), showing no significant difference.

The overall complication rate was 28.6% and the recurrence rate was 5%, with higher rates in the DPS group. Complications occurred in 21.6% of endoscopic procedures compared with 58.3% of other interventions (*p* = 0.028; RR = 0.37, 95% CI 0.18–0.75), indicating a significantly lower risk with the endoscopic approach. Death occurred in 3.9% of endoscopic cases and 8.3% of other interventions (*p* = 0.48); no significant difference was observed.

The mean length of hospitalization was significantly shorter for patients treated endoscopically (7.9 ± 6.4 days) compared with other interventions (17.1 ± 9.0 days, *p* = 0.0048; mean difference = −9.2 days, 95% CI −15.1 to −3.3). Mean hospitalization costs were lower in the endoscopic group (€3.989 ± 2.987) compared with other interventions (€8.732 ± 10.503), although the difference did not reach statistical significance (*p* = 0.15).

## 4. Discussion

This single-center retrospective study compared EUS-guided drainage (LAMS vs. DPS) with percutaneous or surgical drainage for symptomatic peripancreatic fluid collections (PFCs). The analysis focused on clinical efficacy, safety, recurrence, and cost efficacy in a tertiary care setting.

The type of collection was the primary determinant of the chosen technique. Lumen-apposing metal stents (LAMS) were chosen for walled-off necrosis (WON), whereas double pigtail stents (DPS) were predominantly used in pancreatic pseudocysts (PPs). This distribution reflects current practice recommendations, including the European Society of Gastrointestinal Endoscopy (ESGE) guidelines, which supports the use of DPS for simple pseudocysts and LAMS for necrotic collections—particularly when direct endoscopic necrosectomy may be required [16]. Our results align with previously reported success rates for endoscopic management. EUS-guided drainage was reported to be effective in treating PFCs, achieving an overall clinical success rate of 65% with a low recurrence rate of 5%.

Endoscopic necrosectomy was performed in 25% of cases, almost exclusively among patients treated with LAMS, consistent with the higher prevalence of necrosis in this group. The overall complication rate of 28.6% and comparable rates between LAMS (18.5%) and DPS (25%) indicate that both approaches have acceptable safety profiles when performed in experienced centers. No procedure-related mortality was reported, further supporting the safety of minimally invasive endoscopic techniques.

Percutaneous drainage, although effective in providing temporary decompression and symptom relief, achieved only partial or transient improvement in about half of treated cases. Importantly, no WON was primarily managed by percutaneous drainage, underlining the preference for endoscopic management whenever feasible. This finding aligns with the “step-up” approach advocated by ESGE and American Gastroenterological Association (AGA) guidelines, in which percutaneous drainage is reserved for cases with inaccessible collections or contraindications to endoscopy [17,18].

One illustrative case with a step-up approach involved a 57-year-old male with a large WON following acute pancreatitis. Initially, percutaneous drainage performed in a different facility led to incomplete resolution. EUS performed in our clinic showed the persistence of a 7.5/6 cm necrotic collection with thin walls. Subsequent endoscopic drainage (ED) using LAMS, followed by 3 sessions of DEN resulted in complete resolution within one month.

A key factor influencing long-term outcomes in PFCs management is the presence of a disconnected pancreatic duct (DDS), a condition frequently underdiagnosed in clinical practice. In our cohort, 4 patients were diagnosed with DDS, all initially treated by percutaneous drainage. Although transient symptom relief and partial reduction in collection size were achieved, all four required subsequent endoscopic or surgical intervention due to persistent or recurrent fluid collections. This finding highlights a major limitation of percutaneous drainage in the context of DDS: while it provides short-term decompression, it fails to address the underlying ductal disruption, predisposing to recurrence once external drainage is withdrawn.

Disconnected pancreatic duct syndrome (DDS) represents a distinct pathophysiologic entity characterized by complete disruption of the main pancreatic duct with a viable upstream segment that continues to secrete pancreatic juice [19]. In such cases, internal drainage is necessary to redirect secretions into the gastrointestinal tract. EUS-guided transmural drainage, particularly with the placement of long-term indwelling plastic stents, has been shown to offer durable control by maintaining a fistulous tract for physiological outflow [19,20,21].

It seems that percutaneous drainage offers only transient decompression without addressing the source of leakage, leading to high recurrence rates once drains are removed. Conversely, endoscopic transmural drainage—particularly when followed by long-term indwelling plastic stents—provides a route for internal drainage and mitigates recurrence [22]. We found a 5% recurrence rate, higher in the DPS group—but this could hide unrecognized DDS cases, thereby one interesting question arises: could these recurrent cases correspond to undiagnosed DDS rather than true drainage failure?

A step-down approach, transitioning from LAMS to DPS in confirmed DDS, could balance effective cavity resolution with durable ductal decompression. Our experience supports current ESGE and AGA recommendations advocating a tailored, anatomy-based approach to PFCs management. In patients with suspected or confirmed DDS, a step-up strategy—beginning with percutaneous drainage for sepsis control, followed by endoscopic transmural drainage for long-term management—appears most effective. Future protocols should routinely integrate pre-procedural assessment of ductal anatomy and continuity through advanced imaging such as magnetic resonance cholangiopancreatography (MRCP) or pancreatography in order to guide stent selection and duration [23]. Recognition of DDS early in the treatment pathway is essential to prevent recurrence and optimize long-term outcomes. From a cost-effectiveness perspective, although our study found no significant cost difference between LAMS and DPS, recurrence due to unaddressed DDS may lead to repeated interventions, inflating long-term costs.

LAMS were associated with slightly higher costs (€4.546 vs. €3.363) and length of stay (8.4 vs. 7.3 days) compared to DPS, but these differences were not statistically significant. Thus, economic factors alone should not influence the chosen technique. In contrast, clinical context—particularly the nature of the collection and the need for necrosectomy—should guide selection. The substantially higher costs and hospitalization in the percutaneous drainage/surgery group (€8732 ± 10,503, 17.1 ± 9.0 days) further emphasize the efficiency and reduced resource burden of using endoscopic management.

Overall, these results reinforce current international recommendations that favor EUS-guided drainage as the first-line therapy for mature, symptomatic PFCs, with a tailored selection of stent type based on collection characteristics.

A structured step-up strategy and multidisciplinary evaluation involving pancreatologists, surgeons, and interventional radiologists optimize outcomes. Endoscopic step-up approach is associated with better outcomes compared to surgical step-up approaches. The development of innovative endoscopic accessories for necrosectomy improves the results of endoscopic step-up treatment approach. The new concept of Quadrant-Necrosis-Infection (QNI) may also play a role in treatment personalization [17,24].

Our findings reinforce a clinical decision-making paradigm based on anatomical characteristics rather than device preference. In our experience, DPS remains preferable for uncomplicated pseudocysts due to similar efficacy and lower cost, while LAMS should be reserved for WON or when necrosectomy is anticipated. Patients’ safety and favorable clinical outcome should always be the main concern, so particular situations require personalized care. Sometimes a patient may need combined techniques or reinterventions in order to achieve the desired outcome.

Nevertheless, acute pancreatitis complications are complex, and a single treatment strategy cannot be advocated for all patients. The drainage route and treatment strategy depend upon the location of (peri)pancreatic collections, extent of disease and expertise of the center.

Our study presents several limitations. Firstly, its retrospective design means that the data were drawn from records originally collected for clinical rather than research purposes, which may introduce missing or inconsistent information, reduce control over confounding variables, and limit the ability to establish temporality or causality. Secondly, the absence of systematic, extended-duration follow-up restricts the present findings to relatively short-to-mid-term outcomes and precludes reliable assessment of delayed effects, long-term durability, late complications, or relapse beyond the available timeframe.

Indeed, when talking about long-term outcomes, it is worth consideration. Although our study reported a low rate of recurrence (5%), recurrent pseudocysts and stent-related complications such as occlusion or buried-stent syndrome have been reported in prior studies, particularly with LAMS used beyond the recommended time. Patient-reported outcomes (pain relief, quality of life, recovery time) were not particularly assessed in our cohort but represent important endpoints for future prospective research.

## 5. Conclusions

EUS-guided drainage is a safe, effective, and cost-efficient treatment for symptomatic peripancreatic fluid collections. Lumen-apposing metal stents (LAMS) are best suited for walled-off necrosis (WON), facilitating necrosectomy if required. Double pigtail plastic stents (DPSs) remain appropriate for uncomplicated pseudocysts (PPs), proving comparable outcomes and safety with lower device cost. In this cohort, no significant differences were observed between LAMS and DPS regarding the complication rate, hospital stay, or costs, suggesting that clinical factors and not economic considerations should guide the choice of drainage technique.

Percutaneous drainage (PD) offers an alternative route in selected cases but demonstrates limited long-term efficacy and higher overall resource use.

These findings support a tailored, step-up approach, consistent with ESGE and AGA guidelines, prioritizing endoscopic drainage based on the nature of the collection to optimize patient outcomes.

## Figures and Tables

**Table 1:** Baseline characteristics of the patients included.

Variable	Total (N = 63)	LAMS (N = 27)	DPS (N = 24)	Other (N = 12)
Age, years (mean ± sd)	54.8 ± 14.3	53 ± 14.3	59 ± 13.9	50.4 ± 14
Gender, male (%)	44 (69.8%)	17 (62.3%)	17 (70.8%)	9 (75.0%)
Type of collection, n (%)				
Pc	37 (58.7%)	8 (29.6%)	22 (91.7%)	7 (58.3%)
Won	26 (41.3%)	19 (70.4%)	3 (12.5%)	5 (41.7%)
Size of collection, cm (mean ± sd)	11.5 ± 7.2	11.74 ± 3.41	12.29 ± 10.8	9.6 ± 4
Location, n (%)				
Head	6 (9.5%)	3 (11.1%)	3 (12.5%)	0 (0.0%)
Head-body	6 (9.5%)	1 (3.7%)	4 (16.7%)	1 (8.3%)
Body	29 (46.0%)	12 (44.4%)	11 (45.8%)	6 (50.0%)
Body-tail	19 (30.2%)	9 (33.3%)	4 (16.7%)	5 (41.7%)
Tail	3 (4.8%)	1 (3.7%)	2 (8.3%)	0 (0.0%)
Extent of pancreatic necrosis, n (%)				
No necrosis	14 (22.2%)	1 (3.7%)	11 (45.8%)	2 (16.7%)
<30%	14 (22.2%)	9 (33.3%)	3 (12.5%)	2 (16.7%)
30–50%	18 (28.6%)	12 (44.4%)	4 (16.7%)	2 (16.7%)
>50%	9 (14.3%)	4 (14.8%)	0 (0.0%)	5 (41.7%)

**Table 2:** Primary and secondary endpoints.

Variable	Endoscopic Drainage		Other (N = 12)	*p* Value	Relative Risk/Mean Difference (95% CI)
	LAMS (N = 27)	DPS (N = 24)			
Reduction of the collection (≥50%)	20 (74.1%)	18 (75.0%)	3 (25.0%)	0.0022	2.98 (95% CI 1.10 to 8.05)
Need for den (no, %)	14 (51.9%)	0 (0.0%)	2 (16.7%)	0.7140	1.65 (95% CI 0.43 to 6.30)
Complications (no, %)	5 (18.5%)	6 (25.0%)	7 (58.3%)	0.0281	0.37 (95% CI 0.18 to 0.75)
Death (no, %)	0 (0.0%)	2 (8.3%)	1 (8.3%)	0.9999	0.47 (95% CI 0.08 to 27.52)
Recurrence (NO, %)	1 (3.7%)	2 (8.3%)	0 (0.0%)	0.9999	1.47 (95% CI 0.08 to 27.52)
Costs (Euro, mean ± SD)	4545.9 ± 3494.2	3363.3 ± 2196.7	8732 ± 10,503.1	0.1485	−€4742.6 (95% CI −11.448.91 to +1.963.75)
Hospital stay (days, mean ± SD)	8.4 ± 7.2	7.3 ± 5.6	17.1 ± 9.0	0.0048	−9.2 days (95% CI −15.13 to −3.31)

## Data Availability

Data that support the findings of this study and materials are available from the first author upon request.

## Abbreviations

Abbreviation	Full Form
AGA	American Gastroenterological Association
ANOVA	Analysis of Variance
CI	Confidence Interval
DDS	Disconnected Pancreatic Duct Syndrome
DEN	Direct Endoscopic Necrosectomy
DPS	Double Pigtail (Plastic) Stent
ED	Endoscopic Drainage
EUS	Endoscopic Ultrasound
ESGE	European Society of Gastrointestinal Endoscopy
Fr	French (catheter/stent size unit)
LAMS	Lumen-Apposing Metal Stent
LOS	Length of Hospital Stay
MRCP	Magnetic Resonance Cholangiopancreatography
PD	Percutaneous Drainage
PFC(s)	Peripancreatic Fluid Collection(s)
PP(s)	Pancreatic Pseudocyst(s)
RR	Relative Risk
SD	Standard Deviation
SPSS	Statistical Package for the Social Sciences
WON	Walled-Off Necrosis
